# A Critical Review of Modern Concepts for Teeth Whitening

**DOI:** 10.3390/dj7030079

**Published:** 2019-08-01

**Authors:** Matthias Epple, Frederic Meyer, Joachim Enax

**Affiliations:** 1 Inorganic Chemistry and Center for Nanointegration Duisburg-Essen (CeNIDE), University of Duisburg-Essen, Universitaetsstr. 5-7, 45117 Essen, Germany; 2 Dr. Kurt Wolff GmbH & Co. KG, Research Department, Johanneswerkstr. 34-36, 33611 Bielefeld, Germany

**Keywords:** teeth, toothpaste, whitening, peroxides, abrasives

## Abstract

Besides prevention of caries and periodontitis, an increasing number of oral care products focus on teeth whitening. The aim of this review is to summarize and discuss frequently used whitening agents and their efficacy from a chemical viewpoint. Therefore, a comprehensive literature survey on teeth whitening agents and products was conducted. The current whitening methods are analyzed and discussed from a chemist’s viewpoint. Frequently used whitening agents are abrasives (mechanical removal of stains), antiredeposition agents (prevention of deposition of chromophores), colorants (intended to lead to a white color), proteases (degradation of proteins), peroxides (oxidation of organic chromophores), and surfactants (removal of hydrophobic compounds from tooth surface). In-office bleaching using peroxides is effective, but side effects like tooth sensitivity or a damage of the natural organic matrix of enamel and dentin may occur. The applicability of abrasives in teeth whitening is limited due to potential tooth wear, especially when toothpastes with high RDA values are used. The effect of other whitening agents in vivo is often unclear because of a shortage of placebo-controlled clinical trials.

## 1. Introduction

The mineral phase of human teeth consists of calcium phosphate in the form of hydroxyapatite, Ca_5_(PO_4_)_3_(OH) [1,2,3,4,5]. The inner part of a tooth is called dentin, which is a protein-rich bone-like biocomposite containing about 70% hydroxyapatite with proteins (mainly collagen) and water forming the rest [6,7]. Enamel, the outer part of a tooth, is a highly mineralized tissue containing about 97% hydroxyapatite in the form of micrometer-long needles that form a complex hierarchical organized microstructure [5,8]. Its hardness and fracture toughness stem from a complex entanglement of the hydroxyapatite needles that are connected via an organic protein phase. The enamel surface itself is covered by the pellicle, which contains mainly salivary proteins, carbohydrates, and lipids [9,10].

The original color of pure hydroxyapatite (i.e., without substituting foreign ions) is colorless/white, which also broadly holds for the integrated proteins. Consequently, natural enamel has a white color with some translucency. However, due to continuous chemical and mechanical wear of enamel with increasing age (erosion, etc.), the enamel will become thinner and more translucent, i.e., the dentin will become more visible and the overall tooth color will become darker [11].

Furthermore, the “natural” white color of teeth is often compromised due to stains resulting from wine, tea, coffee, smoking, etc. [12]. Whitening formulations for home use (e.g., toothpastes in combination with toothbrushes) and professional use in the dental practice (e.g., bleaching or professional dental cleaning) try to address this problem. In this context, whitening is defined as any means to increase the visual whiteness of a tooth.

The aim of modern oral care products is to prevent caries and periodontitis, which are common challenges of our societies worldwide [13,14,15,16]. Caries and periodontitis can be prevented mainly by tooth brushing with a manual or electric toothbrush in combination with toothpaste as well as a healthy diet (e.g., low sugar intake, no excessive use of erosive drinks) and lifestyle (e.g., no smoking, low levels of stress, not being overweight) [17,18]. Modern toothpastes are highly complex formulations which contain many different agents for the prevention of caries and periodontitis, e.g., fluorides (sodium fluoride, amine fluoride etc.), chlorhexidine, stannous, zinc salts and calcium phosphates such as hydroxyapatite or amorphous calcium phosphates, and surfactants as well as different abrasives for an efficient plaque removal [2,3,15,17,19,20,21,22].

In addition to that, an increasing number of oral care products also (sometimes mainly) focus on teeth whitening. This is due to cosmetic reasons, because many people prefer white teeth and a bright smile as it may also affect their quality of life [23,24]. Lifestyle habits like smoking or consumption of red wine or black tea can lead to darker teeth [25]. Additionally, the tooth color in general also depends on the tooth age [11].

Consequently, oral care companies have introduced many different teeth whitening products. Here we present an overview of common teeth whitening agents and discuss their efficiency as well as potential risks from a chemist’s viewpoint. We believe that this will help both dentists and patients to assess the benefits and the potential risks of whitening treatments. We also hope that some myths (as we would like to call them) that are used to advertise some formulations will be more critically considered after our thorough assessment from a chemical point of view.

## 2. Staining of the Tooth Surface

Colored compounds in the tooth are so-called chromophores, both of organic and of inorganic origin [26,27]. Chromophores absorb light in the visible range and reflect mainly the complementary color that is recognized by the eyes, typically yellow or brownish in the case of teeth. Organic chromophores are small organic molecules like tannins or furfurals, e.g., from coffee, tea, red wine, or fruits. Characteristics of these molecules are double bonds (e.g., carbonyl groups or aromatic groups). Inorganic chromophores are colored transition metal ions like Fe^2+^/Fe^3+^, Cu^2+^, or Mn^2+^. In the form of metal complexes, organic and inorganic chromophores may also be present in combination, e.g., in hemoglobin where a colored porphyrin ligand (organic) is combined with a colored iron ion (inorganic) [26,27].

Stains can be of intrinsic and extrinsic origin [12,26,27]. Intrinsic stains are localized inside the tooth, either in the enamel or in the underlying dentin. They can result from excessive fluoride intake during tooth formation (fluorosis), from tetracycline incorporation, and a number of metabolic diseases and systemic factors during tooth development. The severity of fluorosis, for example, can be classified by Dean’s index, which ranges from questionable, very mild, mild, moderate, and severe [13].

Intrinsic staining of teeth happens prior to tooth eruption during tooth development. However, intrinsic staining can also occur after tooth eruption. Mainly pulpal hemorrhagic products following trauma may lead to intrinsic discoloration by blood penetration into the dentin tubuli [28]. They can also be caused by dental procedures like amalgam fillings or endodontic treatments. As enamel is a solid structure, abrasive techniques are only able to remove intrinsic stains if they remove part of the enamel, i.e., the outermost part of a tooth [29]. However, this cannot be performed as part of the daily oral hygiene at home.

Another option is the use of chemical bleaching agents that penetrate the enamel structure [26,30]. As most enamel discolorations are caused by developmental malformation or incorporation of ions in the natural enamel structure (e.g., fluorosis), bleaching agents will not completely remove them [31]. The removal of intrinsic stains in dentin is almost impossible by any chemical or mechanical means from the outside. Due to the microporous nature of dentin, stains adhere very strongly [32]. Internal treatments are possible, i.e., by endodontic bleaching with peroxides [30], but these procedures are invasive treatments that are performed in dental clinics only [33].

Extrinsic staining is present on the tooth surface, i.e., on enamel and exposed dentin, especially on tooth surfaces which are difficult to clean and on surfaces with a thick pellicle layer [26,29,30,32,34]. Those stains consist of organic and inorganic chromophores that are either directly adsorbed to the tooth (especially if its surface is rough) or (more likely) incorporated into calculus, biofilm and/or pellicle [12]. Chemically, these environments are well suited to host organic and inorganic chromophores. Most organic dyes show a high affinity to proteins, i.e., it is well conceivable that they are present on or inside plaque and pellicle. Calculus as a predominantly inorganic pathological calcification, based on calcium phosphates (i.e., hydroxyapatite (Ca_5_(PO_4_)_3_(OH)), whitlockite (β-(Ca,Mg)_3_(PO_4_)_2_), octacalcium phosphate (Ca_8_(HPO_4_)_2_(PO_4_)_4_·5 H_2_O), and brushite (CaHPO_4_·2 H_2_O)), is able to incorporate other inorganic ions (chromophores) into the calcium phosphate lattice [1,35]. Their origins are usually chromophore-containing foods, beverages, or smoking [25].

In addition to that, ingredients of oral care products themselves may lead to the staining of tooth surfaces. This is called “indirect staining” because these ingredients typically have a different color than the resulting stain [32]. Typical examples include stannous fluoride, SnF_2_, and other stannous salts, as well as chlorhexidine (e.g., in form of mouth rinses), which are widely used as antibacterial agents but may have the side effect of staining the tooth surface, especially after long-term use [32,36,37]. Thus, researchers have proposed alternative anti-biofilm agents that do not stain the tooth surface. Examples include particulate calcium phosphates like hydroxyapatite, which are white powders [3]. In an in situ study, Kensche et al. showed that a hydroxyapatite mouth rinse (without other ingredients than water) reduces the initial bacterial colonization to bovine enamel surfaces similar to 0.2% chlorhexidine. This effect can be explained by anti-adhesive properties of hydroxyapatite particles [38]. While chlorhexidine is known as an antibacterial agent that inhibits the metabolism of bacteria, the staining effect of chlorhexidine is very complex and has been intensively discussed, for example, by Addy and Moran [32].

Notably, chromophores can also be formed by chemical processes (e.g., oxidation) of initially colorless compounds. Colored tin sulfide, SnS, may result from the chemical reaction of stannous fluoride, SnF_2_, from a toothpaste with volatile sulfur compounds produced by oral bacteria. Extrinsic stains can be removed by abrasive techniques (e.g., by toothpastes and toothbrush as well as professional dental cleaning) and also by chemical treatment (e.g., by peroxides) [26,29,30,34].

## 3. Whiteness of Teeth

In order to assess the performance of a whitening agent, in vitro and in vivo methods have been developed to quantify the degree of whiteness and of staining (e.g., the pellicle cleaning ratio [PCR] is a very prominent in vitro approach) [39]. Staining solutions to determine the PCR in vitro often contain coffee or tea in order to imitate a natural staining process in the oral cavity [40]. Several methods can be used to evaluate the tooth color, e.g., a visual assessment using shade guides, spectrophotometry, colorimetry, or computer analysis of digital images [41].

The Lobene stain index is commonly used and is based on a visual inspection of the tooth color [12]. It can only be used to assess extrinsic stains. Staining is classified regarding its intensity (no stain, light stain, moderate stain, and heavy stain) and area (no stain detected, stain covering up to 1/3 of the region, stain covering > 1/3 to 2/3 of the region, and stain covering > 2/3 of the region) [42].

Tooth stain can be semi-quantitatively assessed by color shade guides [12]. A more quantitative assessment of tooth color and brightness requires the measurement of optical reflection spectra as a function of the light wavelength and their interpretation with respect to different colors and their intensity. Quantitative methods are available, based on the CIEL*a*b* color tables, based on lightness (L*), red/green (a*), and blue/yellow (b*). The numbers can be combined in the CIELab equation to give a relative change in tooth color ΔE [26,34,39].

Besides measuring absolute whiteness numbers, the performance of whitening agents is usually assessed in a relative way by comparing the whiteness before and after a treatment. A non-uniform color of a tooth may complicate the analysis. Positioning splints can be used to identify positions in the mouth [26].

## 4. In-Office Teeth Whitening Using Peroxides

To achieve teeth whitening, many different agents are used, e.g., in commercially available toothpastes (Table 1).

Tooth whitening can be performed both by professionals in the dental practice (“in-office”) and at home (over-the counter; “OTC”) by patients themselves. Chemically, bleaching with hydrogen peroxide (H_2_O_2_; H-O-O-H) or calcium peroxide (CaO_2_; Ca^2+ −^O-O^−^) and related compounds are prominent options [12,26,27,34,43].

In-office bleaching (“power bleaching”) is performed with concentrated solutions of H_2_O_2_ in water (typically 35 wt%) for about 20–30 min. Care must be taken because a concentrated hydrogen peroxide solution is highly oxidizing and harmful to soft tissue. Therefore, gingiva and tongue must be protected by suitable means (e.g., rubber dam, water-soaked gauze). In some cases, dental pulp irritation was reported for in-office tooth bleaching [44]. Furthermore, peroxides are antibacterial agents that may lead to an imbalance (dysbiosis) of the oral microbiome [45]. The oxidative action is sometimes supported by irradiation with a heat lamp to enhance the oxidative action [26]. From a chemical viewpoint, this irradiation should not change the oxidative effect of hydrogen peroxide, but it may enhance the reaction rate due to local temperature increase. In 2000, Viscio et al. stated that irradiation for activation of hydrogen peroxide has not been clinically validated so far [26]. In a clinical study, no significant effect of light irradiation during the application of 35% hydrogen peroxide was found [46]. Carey stated in the year 2014 that there is still no proven effect of an irradiation, neither for the amount of whitening achieved nor for the persistence of the whitening treatment during the bleaching process [27].

Overnight (“nightguard”) bleaching is accomplished by application of a 10–20% carbamide peroxide-containing gel (see below) in a patient-specific mouthguard [26]. A 10% carbamide gel has been approved by the American Dental Association for home bleaching [30,43]. Due to the lower concentration of hydrogen peroxide, a number of overnight treatments are necessary to achieve visible effects [30]. The whitening effect of both power bleaching and nightguard bleaching was reported to persist for several years after treatment [27,30]. Other bleaching options are paint-on gels and whitening strips, both based on peroxides [27,43].

Note that hydrogen peroxide decomposes over time, especially in the presence of catalytically active compounds like metal ions, noble metals (Pt), and enzymes (catalase), according to

(I) H_2_O_2_ → H_2_O + ½ O_2_.

The released oxygen molecule can act as an oxidant. However, the normal oxidative action of hydrogen peroxide depends on the pH value:

(II) H_2_O_2_ + 2 H^+^ + 2 e^−^ → 2 H_2_O, or
(III) H_2_O_2_ + 2 e^−^ → 2 OH^−^.

This also happens after hydrolysis of peroxide-precursor compounds that release hydrogen peroxide:

(IV) MgO_2_ + 2 H_2_O → Mg(OH)_2_ + H_2_O_2_ (magnesium peroxide);
(V) CaO_2_ + 2 H_2_O → Ca(OH)_2_ + H_2_O_2_ (calcium peroxide);
(VI) Na_2_(CO_4_) + H_2_O → Na_2_CO_3_ + H_2_O_2_ (sodium percarbonate);
(VII) CO(NH_2_)_2_·H_2_O_2_ → CO(NH_2_)_2_ + H_2_O_2_ (carbamide peroxide, an adduct of urea and hydrogen peroxide, contains about 36 wt% hydrogen peroxide).

Chemically, the conjugated systems of unsaturated organic compounds (like aromatic compounds, alkenes, alkynes) that absorb light in the visible spectrum and therefore act as chromophores are oxidized by peroxides so that light is no longer absorbed. The underlying chemical reactions are manifold and complex. In short, bleaching with peroxides leads to the oxidation of organic chromophores to non-colored organic compounds. It is tacitly assumed that these organic compounds are removed from the tooth surface by subsequent washing steps. Notably, inorganic ions like Fe^3+^ are not oxidized by peroxides and remain colored after the treatment. However, it is chemically reasonable to assume that they are also removed after surrounding (bio-)organic molecules have been destroyed by oxidation so that the ions are released into the surrounding bleaching liquid [26,30]. The overall kinetics of these complex chemical reactions are not known. Consequently, Fearon has stated that the degree of whitening can be only insufficiently controlled during power bleaching [30].

## 5. Whitening Toothpastes

Dedicated whitening toothpastes are on the market, e.g., for smokers [12]. Additionally, also many “multifunctional” or “all-in-one” toothpastes claim whitening effects. They often contain special abrasives and/or whitening agents (see Table 1).

To analyze the efficiency of whitening toothpastes, in vitro (usually on extracted human teeth or animal teeth) and in vivo studies (usually clinical trials) have been performed [12,17]. For whitening toothpastes, we also distinguish between (external) stain prevention and (external) stain removal.

Abrasives are the most important ingredients in toothpaste formulations for an efficient stain removal [17]. Whitening toothpastes often (but not always) contain harder abrasives and a higher amount of these than conventional toothpastes to achieve a sufficient abrasion of external stains (Table 2) [12,26,29,47]. In general, a toothpaste with a high abrasivity will remove the outer part of the enamel, including the attached and the incorporated stains. The demand for a high polishing action (with high whitening effect) is constrained by the potential damage to the outer tooth layer (enamel and exposed dentin).

Thus, the abrasivity of a toothpaste is limited by a potentially harmful action on the enamel, exposed dentin, and gingiva by compounds that are too abrasive. In contrast, a toothpaste with low abrasivity (e.g., for sensitive teeth; gentle cleaning of exposed dentin) may lead to increased staining of the tooth surface because of lower cleaning efficacy. Common abrasives are hydrated silica, SiO_2_·n H_2_O, calcium carbonate, CaCO_3_, and alumina, Al_2_O_3_ (Table 2). Additionally, these abrasives may vary in particle size, morphology, and hardness [29,47]. Especially, the properties of silica abrasives strongly depend on various parameters such as water content, cross-linking, particle shape, and particle size [47].

Particulate hydroxyapatite is a biomimetic agent used in preventive oral care [2,15,19,20,38,48,49]. Additionally, it also appears as promising abrasive due to its similarity to tooth minerals, besides a whitening effect that is not only due to polishing but also to its presence on the tooth surface [50]. It was shown that hydroxyapatite particles attach to the enamel [38,51]. However, a low concentration of hydroxyapatite particles leads to a lower surface coverage of the tooth [52]. Other white pigments (e.g., TiO_2_) show a higher refractive index than calcium phosphates, but are not biomimetic. Another application form is a coating of the teeth with calcium phosphate in the form of a polymer-based gel or by using high concentrations of hydroxyapatite [52].

Dabanoglu et al. have reported an in vitro study with particulate hydroxyapatite and obtained good whitening results on extracted human premolars [53]. Jin et al. have reported some whitening effects of various calcium phosphate particles in a toothpaste that also contained carboxymethylcellulose on extracted human teeth [54]. Scanning electron micrographs in both publications did not indicate a dense layer of calcium phosphate particles on the tooth surface. Hence, the reason for the whitening effect is basically unknown [53,54]. Kim et al. reported that the whitening effect of a hydroxyapatite-based toothpaste was comparable with two commercial toothpastes, but the full composition of the toothpastes was not given [55]. Bommer et al. reported on a combined in vitro/in vivo study on nanohydroxyapatite and a self-assembling peptide matrix as a glue to the tooth surface and observed a significant whitening effect that they ascribed to diffuse reflection by hydroxyapatite particles on the tooth surface [56]. Lelli et al. demonstrated how a zinc-carbonate hydroxyapatite-containing toothpaste can lead to particle adsorption on the tooth surface [51]. Fabritius-Vilpoux et al. quantitatively studied the interaction between teeth and particulate hydroxyapatite in vitro and showed a dose-response relationship [52].

Much effort is directed to developing abrasives that remove stains with a minimal damaging effect on the enamel [47]. The RDA value (radioactive dentin abrasion) is an accepted way to express the abrasive performance of toothpastes. Toothpastes with RDA values below 250 are generally accepted as safe. A means to assess the cleaning effect of toothpastes and abrasives is the PCR value (pellicle cleaning ratio) [12]. In general, a high RDA value is associated with a high PCR value as both parameters are based on the same effect (mechanical abrasion on the tooth surface). However, the mathematical correlation between both parameters is not very pronounced [19,26,47,57]. Furthermore, the abrasiveness of toothpaste formulations is also affected by many other factors like toothbrush filaments, etc.

Bleaching compounds in whitening toothpastes are also peroxide-based. Due to the chemical instability of hydrogen peroxide, other compounds like calcium peroxide, sodium percarbonate, and magnesium peroxide are used. After hydrolysis, they release H_2_O_2_ that will then exert its bleaching action. From a chemical point of view, it is clearly important to assure that the shelf life of this bleaching compound is sufficiently long because (in most cases) toothpastes are also water-based formulations.

Bleaching compounds in cosmetics, such as toothpastes, are legally regulated due to their potential harms. In particular, due to safety concerns, the concentration of hydrogen peroxide in cosmetic toothpastes is usually limited to about 1 wt%; in the European Union it is limited to a maximum of 0.1 wt% [26,29]. From a chemical viewpoint, it is questionable whether such a low peroxide concentration will lead to a sufficient oxidizing power in a toothpaste. This is based on the inherent instability of hydrogen peroxide in an aqueous formulation and on the short contact time during toothbrushing (about 2–3 min) and the additional dilution due to salivary flow [29]. Activating agents like transition metal ions have been proposed to enhance the oxidative capacity of hydrogen peroxide [26]. It has been stated by Demarco et al. that whitening toothpastes work only by their abrasive effect and not by bleaching [43]. In contrast, Joiner has cited a number of studies where whitening toothpastes containing around 1% peroxide have shown a significant whitening effect. However, only in vitro studies were discussed [12]. Another study states that the efficacy of several whitening-agents used in toothpastes, such as peroxides, is debatable [29].

Besides peroxides, many other whitening agents are commercially available and used in oral care products. This includes, surface active agents (surfactants), antiredeposition agents, colorants, enzymes, and polyaspartate [12,17,26].

Surfactants remove hydrophobic compounds from the tooth surface by solubilization as in cleaning applications (soap, washing powder etc.) [12,29]. Frequently used surfactants in toothpaste formulations are e.g., sodium lauryl sulfate (SLS) and cocamidopropyl betaine [17].

Antiredeposition agents are expected to prevent the subsequent deposition of chromophores after the whitening procedure [26,29]. They also act as calculus control agents that inhibit the formation of calculus where external stains may be incorporated [29]. Polyphosphates and sodium citrate are typical antiredeposition components. They are intended to prevent the formation of calculus, which is basically a pathological deposition of calcium phosphate phases from saliva [8,58,59]. It is assumed that they have a strong adsorption affinity to the tooth surface. They can also bind colored inorganic ions and prevent their deposition [26]. The efficiency of antiredeposition components in whitening toothpastes has been demonstrated [60]. Pyrophosphate, sodium trimetaphosphate (STP), and sodium hexametaphosphate (HMP) are typical examples of substances that are used in whitening toothpastes with proven performance [12,29]. A relatively new approach is the use of polyaspartate in whitening formulations. A toothpaste with sodium polyaspartate showed better stain preventing properties than a control toothpaste in vivo [9]. Sodium polyaspartate contains many carboxy groups. These may lead to an interference either with the tooth-surface or with the pellicle, leading to a different color-absorption and reflection.

Colorants are specific dyes that are intended to give the teeth a white color. It is yet unclear whether they will attach to the teeth after brushing [26]. However, papers reported the successful application of novel whitening toothpastes containing the blue dye covarine that shifts the reflected color of teeth from yellow into the blue region, thus leading to a whiter appearance [12,61].

Enzymes (proteases) which selectively degrade proteins by hydrolysis of peptide bonds may be a potential approach as future whitening agents [26]. An example is the use of the protease enzyme papain. Papain consists of 212 amino acids and can be isolated from papayas [62]. Joiner discussed the use of papain with conflicting clinical results [12]. Whitening toothpastes with papain haven been combined with bromelain extracts [63]. Papain and bromelain are both cysteine proteases (endopeptidases). This means that they have a cysteine in their active site that covalently attaches to target proteins for subsequent cleavage [64]. By protein degradation, the characteristics (absorption maximum, extinction coefficient) of their chromophores can be changed, leading to less light absorption in the visible range. Lysozymes and peroxidases have also been used in toothpastes [65]. However, when using enzymes, special emphasis must be given to the storage conditions. Most enzymes are only stable for a short time at room temperature. Even during the production process and at the transport and storage temperatures, an inactivation may occur.

Furthermore, an increasing number of toothpastes with activated charcoal are commercially available. Activated charcoal is a nanocrystalline form of carbon with a high specific surface area (>1000 m^2^ g^−1^) and a high number of pores in the nanometer range. It can be prepared by heating organic materials like wood or coal. Activated charcoal is typically used as an adsorbent in different applications. The application of activated charcoal in whitening toothpastes, however, has not yet been studied in detail [66]. A recently published in vitro study compared toothpastes of different manufactures containing different whitening formulations, i.e., with activated charcoal, blue covarine, hydrogen peroxide, or polyethylene microbeads. It was concluded that the toothpastes with polyethylene microbeads and blue covarine were most effective in teeth whitening [66].

In addition to whitening toothpastes, whitening mouth rinses, whitening stripes, whitening dental floss, and whitening chewing gums have been reported in the literature. Whitening mouth rinses containing a low concentration of hydrogen peroxide (1.5%) and sodium hexametaphosphate have been applied with moderate success [43]. Furthermore, mouth rinses do not contain any abrasives, i.e., the stain removal properties are generally inferior to a toothpaste. Whitening dental floss has been brought to the market (coated with abrasive silica), but up to 2009, no clinical report was published [43]. Whitening chewing gum, containing sodium hexametaphosphate, did not show a better performance than normal chewing gum [43].

The clinical efficiency of whitening toothpastes is controversially debated in the literature. Walsh et al. reported a clinical study where a standard toothpaste was compared to a dedicated whitening toothpaste. The effect after 1, 4, and 6 weeks was not very pronounced and within the statistical error margin [67]. Demarco et al. noted that, “Although whitening toothpastes can prevent extrinsic tooth stains, the whitening effect obtained seems not to be clinically significant,” and gave references for this statement [43]. Da Silva et al. performed a cyclic smoke incubation/brushing of bovine tooth discs for seven weeks. Neither the whitening toothpastes nor a conventional toothpaste were able to prevent the staining of the tooth surface [25]. Imfeld and Sener suggested that the term “whitening” should be defined in more detail in order to differ between “normal” and whitening toothpastes [68].

## 6. Potential Risks of Teeth Whitening Concepts

In teeth, an inorganic mineral (calcium phosphate in the form of hydroxyapatite) is combined with an organic protein matrix. Only the chemical and structural interplay between these two components leads to the extraordinary mechanical properties of teeth with respect to hardness and fracture toughness. Thus, teeth are not simply inorganic materials but highly optimized and complex organic-inorganic biocomposites. If aggressive bleaching agents like hydrogen peroxide are applied in high concentrations, it will also damage the organic matrix in the tooth, especially in dentin. We note that enamel contains about 1% organic matrix and dentin contains about ca. 20% organic matrix, mainly collagen [8]. This could lead to a mechanical weakening of the tooth due to a decreasing integration of the calcium phosphate crystals. Fearon discussed a number of studies of different whitening procedures and concluded that changes in the tooth surface structure and increased tooth sensitivity can occur, especially if highly concentrated hydrogen peroxide solutions are applied. There are also reports about a structural damage of enamel surface prisms after application of 35% carbamine peroxide [30]. The risk of adverse effects will increase with the peroxide concentration. In contrast, a chemical reaction between calcium phosphate and peroxides is very unlikely.

An increased tooth sensitivity (bleaching sensitivity) after power bleaching, which persisted a few days, has also been reported [30,69,70,71]. Bleaching sensitivity often occurs after bleaching because small microscopic defects and subsurface pores are caused by the whitening agents (peroxide). This sensitivity is caused by reversible pulpitis, leading to thermal tooth sensitivity [72]. A possible strategy to reduce tooth sensitivity after bleaching is the use of oral care products containing particulate hydroxyapatite [73,74,75,76,77] or potassium nitrate [78]. Clearly, the factors “white teeth” (with a positive influence on the quality of life) and “tooth sensitivity” (with a negative influence on the quality of life) are related [23].

Strong (hard) abrasives like perlite and alumina have a higher hardness than hydroxyapatite (i.e., the tooth mineral) and may not only damage enamel and exposed dentin, but also the gingiva during stain removal, especially when high pressure is applied during tooth brushing. The RDA value, in general, only gives limited information as it measures the abrasion of dentin and not of the much harder enamel. The correlation between PCR and RDA is low [47], but nevertheless exists, therefore a compromise must always be sought between a good stain removal efficiency and the protection of teeth and gingiva.

It is unclear whether bleaching leads to a long-term damage of the teeth due to oxidation of the constituting organic molecules. This damage may affect the mechanical integrity of a tooth (enamel and dentin). Additionally, enhanced tooth sensitivity (bleaching sensitivity) is a common side effect following the use of peroxides [69,71,79].

## 7. Conclusions

For whitening, two major approaches can be distinguished, as follows: Chemical bleaching by peroxides and mechanical cleaning by toothpaste abrasives. Chemical bleaching leads to good results, especially when it is performed with high peroxide concentrations in a controlled environment, i.e., in the dental practice. Mechanical cleaning relies on suitable abrasives that are harder than stains but less hard than enamel. Considerable progress into this direction has been achieved with silica toothpaste formulations in the last years (optimized RDA/PCR ratio) [47,80], but current formulations always represent a compromise between desired cleaning efficiency and unwanted tooth abrasion.

It is not always easy to interpret the results of in vitro studies and clinical studies in the field of teeth whitening. They are not always randomized and, often, more than one parameter is changed. For instance, an enzyme-containing toothpaste should ideally be compared to exactly the same product without the enzyme (placebo). However, for practical reasons, new formulations with different abrasives, additives, etc., are often compared to toothpastes on the market. The simultaneous change of more than one parameter makes definite conclusions difficult or even impossible. It is often not clear which agent in a toothpaste formulation contributes to the whitening effect and to what extent. This should be addressed in further studies by comparing whitening agents separately, i.e., a non-abrasive gel formulation with enzymes vs. the same gel formulation with colorants. Otherwise, there will always be synergetic effects of different whitening agents because toothpastes are complex formulations (abrasives, enzymes, surfactants, etc.) causing an equally complex response in the mouth [17]. The whitening market has a large economic volume, i.e., the competition between manufacturers is high, and not all claims are based on solid scientific evidence. In general, a great challenge for planning and conducting clinical studies in the field of teeth whitening is the selection of population-representative inclusion criteria as teeth staining is strongly correlated to the diet and other factors (like smoking, chlorhexidine).

Finally, the modes of action of many whitening agents in vivo are still unknown. Thus, mechanistic studies are required to understand the mechanisms of action from a chemical and biological viewpoint, which is an important requirement for the development of more efficient teeth whitening formulations. Besides whitening of natural teeth (see above), another important research field is the prevention and removal of stains on restauration materials, e.g., of polymer-based composites [81,82,83,84].

## Figures and Tables

**Table 1:** Examples of commonly used whitening agents in products for home and professional use (in alphabetical order; the most efficient whitening agents are underlined) [12,17].

Whitening Agent	Mode of Action
Abrasives (e.g., hydrated silica, perlite, alumina) → Most important toothpaste ingredient for stain removal	Mechanical removal of extrinsic stains
Antiredeposition agents (e.g., polyphosphates, sodium citrate)	Prevention of the deposition of chromophores and inhibition of calculus formation where external stains could be incorporated
Calcium phosphates (e.g., hydroxyapatite)	Adhesion of white calcium phosphate particles on the tooth surface, and prevention of bacterial attachment/plaque-formation on the teeth
Colorants (e.g., blue covarine)	Shifting color absorption and reflection spectra from yellow to blue
Enzymes/proteases (e.g., papain, bromelain)	Support stain removal due to degradation of proteins (hydrolysis of peptide bonds)
Peroxides (e.g., hydrogen peroxide, calcium peroxide)	Oxidation of organic chromophores
Polyaspartate (e.g., sodium polyaspartate)	Inhibition of plaque-formation
Surfactants (e.g., sodium lauryl sulfate)	Removal of hydrophobic compounds from the tooth surface

**Table 2:** Overview of commonly used abrasives in toothpastes [47]. Hard abrasives remove stains more efficiently than soft abrasives; however, they may be harmful to the enamel and specially to exposed dentin (INCI: International Nomenclature of Cosmetic Ingredients).

Name (INCI)	Chemical Formula	Relative Hardness	Expected Stain Removal
Sodium bicarbonate	NaHCO_3_	Soft	Low
Dicalcium phosphate dihydrate (brushite)	CaHPO_4_ ∙ 2 H_2_O	Soft	Low
Calcium carbonate	CaCO_3_	Soft	Low
Calcium pyrophosphate	Ca_2_P_2_O_7_	Medium hard	Medium
Hydroxyapatite	Ca_5_(PO_4_)_3_(OH)	Medium hard	Medium
Hydrated silica	SiO_2_ ∙ n H_2_O	Medium hard	Medium
Perlite	A mineral silicate	Hard	High
Alumina	Al_2_O_3_	Hard	High
